# Management of Pulmonary Embolism With Thrombus in Transit: A Case Series and an Updated Clinical Insight Review

**DOI:** 10.7759/cureus.79982

**Published:** 2025-03-03

**Authors:** Guillermo Cueto-Robledo, Ernesto Roldan-Valadez, Dulce-Iliana Navarro-Vergara, Marisol Garcia-Cesar, Maria-Berenice Torres-Rojas

**Affiliations:** 1 Cardiorespiratory Emergencies, Hospital General de México "Dr. Eduardo Liceaga", Mexico City, MEX; 2 Research, Instituto Nacional de Rehabilitacion (INR) "Luis Guillermo Ibarra Ibarra", Mexico City, MEX

**Keywords:** anticoagulation, cit management, clot-in-transit, imaging techniques, pe treatment, pulmonary embolism, right heart thrombus, surgical thrombectomy, thrombolysis, venous thromboembolism

## Abstract

Clot-in-transit (CIT) refers to a thrombus temporarily lodged in the right heart chambers, representing a critical and rare complication of venous thromboembolism, particularly in patients with acute pulmonary embolism (PE). This condition poses a significant risk of morbidity and mortality. This report provides a comprehensive overview of CIT in the context of PE, focusing on its definition, etiopathogenesis, risk classification, clinical manifestations, imaging findings, and treatment options. A retrospective review of CIT cases in PE patients at our institution was conducted, complemented by a detailed literature review. Data were analyzed to highlight the clinical findings, imaging results, and diverse treatment strategies employed. Five cases of CIT associated with PE are presented, illustrating varied risk factors, clinical presentations, and imaging findings. Treatment modalities included anticoagulation, thrombolysis, and surgical thrombectomy. Each case underscores the diagnostic challenges and management complexities inherent to CIT. CIT is a life-threatening complication of pulmonary thromboembolism. Early identification and individualized treatment are essential for improving outcomes. This case series provides valuable insights into CIT management and emphasizes the importance of multidisciplinary approaches for optimal patient care.

## Introduction

Pulmonary embolism (PE) is a life-threatening condition with substantial morbidity and mortality [1]. Among its severe manifestations is clot-in-transit (CIT), a rare and critical phenomenon in which a thrombus temporarily resides in the right heart chambers during its transit to the pulmonary arteries [2]. First described by Chapoutot et al. in 1966, CIT is associated with a particularly high risk of mortality, reaching up to 45% if untreated [3]. The unique challenges of diagnosing and managing CIT are compounded by its high mortality risk, which has been emphasized in recent case studies on CIT complicated by deep vein thrombosis (DVT) and pulmonary embolism [4,5]. Although CIT has been increasingly recognized due to advancements in imaging technologies such as echocardiography and computed tomography pulmonary angiography (CTPA), its rarity and lack of comprehensive data pose challenges to clinicians [1].

CIT can originate from systemic veins and is often associated with underlying risk factors such as venous thromboembolism, atrial fibrillation, and structural cardiac anomalies. Most thrombi arise from the lower extremities, emphasizing the importance of identifying associated risk factors for accurate diagnosis and management. Despite its critical nature, CIT remains understudied, with most evidence derived from isolated case reports and small series, highlighting a need for further investigation [6,7].

This case series aims to provide a detailed examination of CIT in the context of PE, focusing on clinical presentations, diagnostic challenges, and treatment strategies. By analyzing cases from our institution and integrating findings from the literature, this report seeks to enhance the understanding of CIT and inform clinical decision-making to improve patient outcomes.

## Case presentation

**Figure 1:**
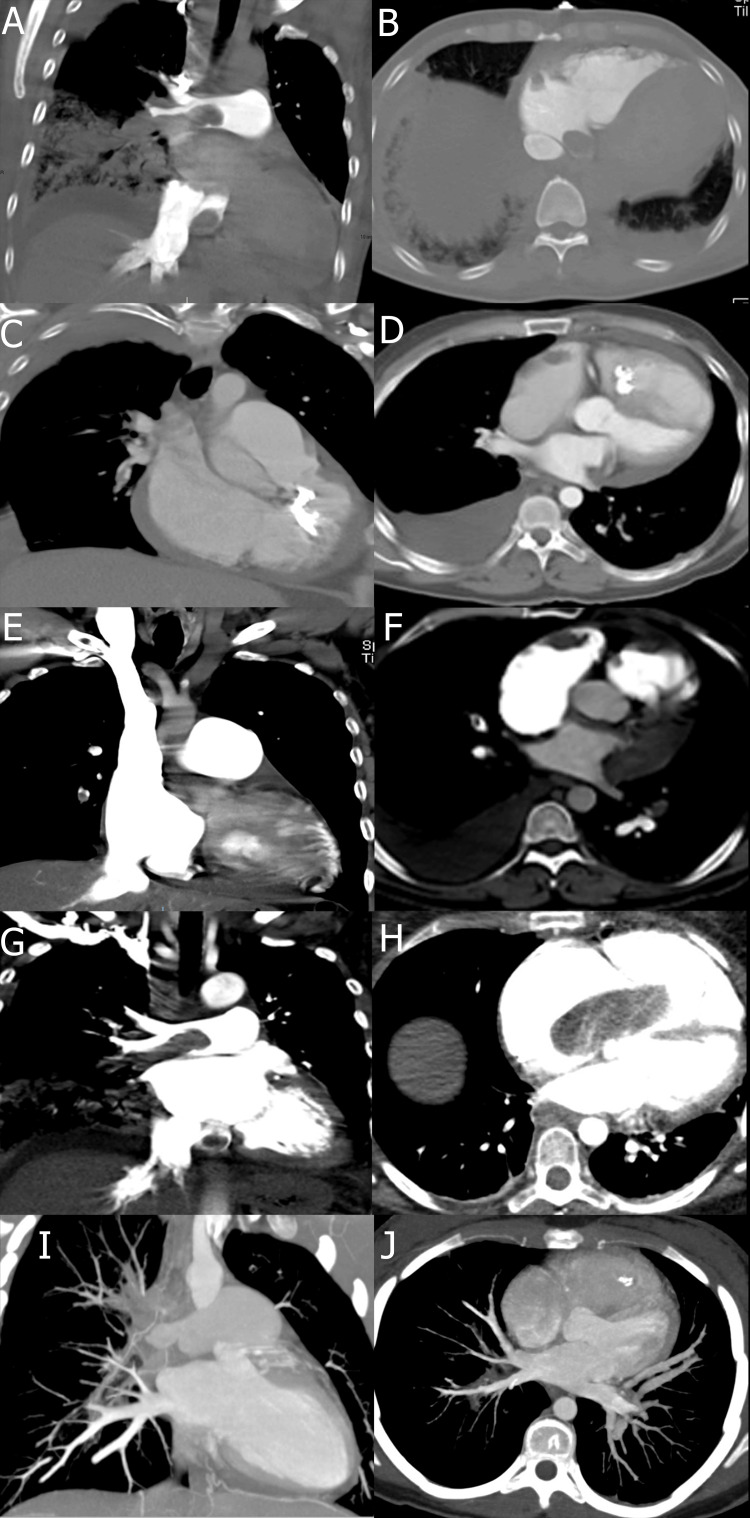
Imaging Findings of Computed Tomography Pulmonary Angiography (CTPA) in Patients With Pulmonary Embolism (PE) and Clot-in-Transit (CIT). Computed tomography pulmonary angiography (CTPA) images demonstrating clot-in-transit (CIT) in five patients with pulmonary embolism (PE). Panels A and B show hypodense filling defects in the right pulmonary artery and right atrium, indicative of mobile thrombi (patient 1). Panels C and D highlight thrombi in the right atrium and ventricle (patient 2). Panels E and F display embolic thrombi extending from the pulmonary arteries into the right atrial appendage (patient 3). Panels G and H illustrate a large, partially occlusive thrombus at the atrioventricular junction (patient 4). Panels I and J show heterogeneous filling defects within the right ventricle, suggestive of varying thrombus morphology (patient 5). Patients are mentioned as per Table 1 and Table 2.

This case series includes five patients diagnosed with CIT in the context of PE, evaluated over an 11-month period. The cohort consisted of three female and two male patients, with a mean age of 34.4 years. The patients presented with a variety of symptoms, including dyspnea (four patients) and hemoptysis (one patient). One individual had a history of primary renal neoplasia, and two were diagnosed with antiphospholipid syndrome (APS). Figure 1 and Table 1 support the diagnostic classifications and clinical findings discussed.

**Table 1:** Clinical Characteristics and Laboratory Findings of Patients With Pulmonary Embolism (PE) and Clot-in-Transit (CIT). This table summarizes the demographic, clinical, and laboratory characteristics of five patients diagnosed with CIT and PE. Notably, patient 1 presented with extensive thrombi involving both atria and ventricles, necessitating surgical embolectomy, while patients 2 and 5 had antiphospholipid syndrome (APS), influencing their anticoagulation strategy. The variation in symptoms and biomarker levels underscores the heterogeneity in CIT presentations. BMI, body mass index; BNP, B-type natriuretic peptide; mEq/L, milliequivalents per liter; mg/dL, milligrams per deciliter; ng/dL, nanograms per deciliter; pg/mL, picograms per milliliter; VTE, venous thromboembolism; y, years

Variable	Patient 1	Patient 2	Patient 3	Patient 4	Patient 5
Age/sex	53 y/female	51 y/male	29 y/male	24 y/female	15 y/female
BMI	19.3	22.7	27.54	28.36	22.3
Risk factors for VTE	Right heart failure	Heart failure, hypothyroidism, and APS	Heart failure	Kidney carcinoma	APS
Symptoms	Dyspnea	Dyspnea, fatigue, and palpitations	Hemoptysis	Dyspnea, fatigue, and syncope	Dyspnea
Risk stratification	Intermediate-high	Intermediate-high	Intermediate-high	Intermediate-high	Intermediate-high
Leukocytes	17.1 × 109/L	6 × 109/L	10.7 × 109/L	11.2 × 109/L	8.5 × 109/L
Hemoglobin	13.2 g/dL	13.6 g/dL	15.4 g/dL	16.6 g/dL	12.2 g/dL
Sodium	130.6 mEq/L	135.9 mEq/L	144 mEq/L	141 mEq/L	135 mEq/L
Potassium	4.8 mEq/L	4.2 mEq/L	4.1 mEq/L	4.4 mEq/L	3.3 mEq/L
Total bilirubin	1.5 mg/dL	1.98 mg/dL	1.97 mg/dL	2.03 mg/dL	0.84 mg/dL
D-dimer	13340 ng/dL	1954 ng/dL	24943 ng/dL	6629 ng/dL	518 ng/dL
Troponin I	354 ng/mL	193 ng/mL	822 ng/mL	309 ng/mL	159 ng/mL
BNP	3198.5 pg/mL	1216 pg/mL	2470 pg/mL	2690 pg/mL	790 pg/mL
Creatinine	2.31 mg/dL	1.03 mg/dL	1.05 mg/dL	1.3 mg/dL	0.9 mg/dL

**Table 2:** Correlation of Pulmonary Embolism Severity Index (PESI) and Bova Scores With Thrombus Location and Clinical Outcomes in Patients With PE and CIT. This table correlates pulmonary embolism (PE) severity scores (PESI/Bova) with thrombus location and treatment outcomes. All patients were categorized as intermediate-high risk, supporting the need for aggressive intervention. The mortality observed in patient 1 underscores the potential severity of CIT when extensive thrombi are involved. RA, right atrium; LV, left ventricle; LA, left atrium; RV, right ventricle; CIT, clot-in-transit

Variable	Patient 1	Patient 2	Patient 3	Patient 4	Patient 5
PESI/Bova	IV/III	III/III	IV/IV	III/IV	IV/IV
Location of thrombi	RA and LV	RA and LA	RV	RA and RV	RV
Treatment	Surgical embolectomy	Anticoagulation	Systemic thrombolysis	Anticoagulation	Systemic thrombolysis
Actual state	Dead	Alive	Alive	Alive	Alive

Risk stratification scores classified all five patients as intermediate-high risk based on the Pulmonary Embolism Severity Index (PESI) (Table 2).

Imaging studies played a pivotal role in diagnosis. Computed tomography pulmonary angiography (CTPA) diagnosed CIT in all cases and revealed bilateral pulmonary involvement in 100% of patients (Figure 1). In 40% of the cases, additional findings, such as pulmonary infarction and pleural effusion, were observed, emphasizing the complexity of the presentations.

Treatment strategies were individualized based on the patient's condition and thrombus characteristics. Two patients underwent systemic thrombolysis using alteplase (patients 2 and 4), while two others (patients 3 and 5) received direct oral anticoagulants (DOACs). One patient, who presented with thrombi in both the right and left heart chambers, underwent surgical embolectomy. Despite aggressive treatment, this patient succumbed to complications related to acute lung injury (patient 1). The remaining four patients were discharged on long-term oral anticoagulants and are alive at the time of follow-up (patients 2, 3, 4, and 5).

Table 1 summarizes the clinical characteristics, risk factors, and laboratory findings of the patients, while Table 2 highlights the correlation between PESI scores, thrombus locations, and treatment outcomes. This detailed case presentation underscores the importance of imaging and multidisciplinary approaches in the management of CIT and PE.

## Discussion

Literature search approach

A comprehensive literature search was conducted using PubMed, Scopus, and Web of Science databases to identify studies focusing on CIT in the context of PE. The search terms included "clot-in-transit," "pulmonary embolism," "anticoagulation," "thrombolysis," and "surgical embolectomy." Articles were included if they reported on diagnostic strategies, imaging findings, or management outcomes related to CIT. Exclusion criteria encompassed studies involving pediatric populations or cases unrelated to PE-associated CIT. This search facilitated the contextualization of our findings within the existing literature.

Imaging and classification

CTPA remains the cornerstone for diagnosing CIT due to its high sensitivity and specificity (Table 3).

**Table 3:** Treatment Modalities for Clot-in-Transit (CIT) Management: Advantages and Disadvantages. This table compares different treatment options for CIT, including anticoagulation, systemic thrombolysis, endovascular therapies, and surgical embolectomy, outlining their advantages and disadvantages to inform clinical decision-making (modified from Igwilo et al. [8]). TEE, transesophageal echocardiogram; RA, right atrium; RV, right ventricle; IVC, inferior vena cava; PA, pulmonary artery; PFO, patent foramen ovale; PE, pulmonary embolism

Treatment	Advantages	Disadvantages
Anticoagulation (AC)	Available in all centers. All patients need AC if there are no contraindications. No special equipment/expertise is needed. Case reports of successful outcomes with only AC	Bleeding complications. Cannot be used if there are absolute contraindications. Increased risk of distal embolization. Higher mortality if used alone in patients who need aggressive care
Systemic thrombolysis	Available in most/all centers. It can be administered rapidly at the bedside, especially in decompensating patients. No special equipment/expertise is needed. Lower mortality rate compared to anticoagulation alone	Cannot be used if there are absolute/relative contraindications. Bleeding complications. May not be effective for chronic clotting. Risk of distal embolization
Endovascular therapies (Inari FlowTriever system)	Useful for CIT in RA, RV, IVC, and PA. FDA-approved	Need guidance with TEE or intracardiac echocardiography. Procedural blood loss due to suction/aspiration. Bleeding complications as patients need anticoagulation
Endovascular therapies (AngioVac system)	Less procedural blood loss due to the reinfusion cannula. FDA-approved	More invasive, as it requires a veno-venous extracorporeal bypass circuit. Need for general anesthesia and perfusionist. Need guidance with TEE or intracardiac echocardiography. Not ideal for RV/acute PE. Only for RA/IVC clots. Bleeding complications as patients need AC
Endovascular therapies (Indigo Aspiration System, Penumbra)	Not approved by the FDA for CIT. Smaller cannula size	Associated with procedural blood loss because there is no reinfusion cannula. Limited availability
Surgical embolectomy	Definitive therapy. Useful if thrombolysis is contraindicated or ineffective. It is useful if patients have open PFO or CIT trapped in PFO	Much more invasive. Limited to tertiary/quaternary centers

**Figure 2:**
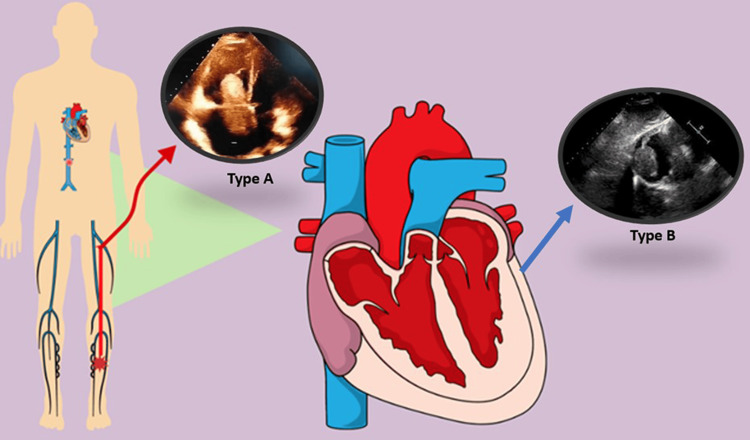
Classification of Right Heart Thrombi According to the European Working Group on Echocardiography Criteria. Type A thrombi are thin, highly mobile, and often associated with acute PE, presenting an increased embolic risk. Type B thrombi are nonmobile and often mural, frequently linked to structural cardiac abnormalities or atrial fibrillation. Type C thrombi resemble myxomas but exhibit high mobility. In this case series, all patients presented with Type A or Type B thrombi, which influenced their respective treatment approaches. The authors designed this figure using a paid subscription to the Mind the Graph online platform (https://mindthegraph.com/), which granted permission for publication. PE: pulmonary embolism

Echocardiography, including transthoracic echocardiogram (TTE) and transesophageal echocardiogram (TEE), serves as a valuable adjunct by visualizing thrombus mobility and morphology. Recent studies highlight the complementary roles of CTPA for precise pulmonary artery visualization and echocardiography for the real-time assessment of thrombus mobility, supporting a dual-modality approach for CIT detection [9,10]. The European Working Group on Echocardiography classification divides CIT into three types: Type A, mobile thrombi originating from peripheral veins; Type B, mural thrombi often associated with atrial fibrillation or cardiac structural abnormalities; and Type C, which mimic myxomas [11]. In this series, patients did not undergo an echocardiogram as it was unavailable; however, all cases were classified as Type A or Type B thrombi using CTPA, which guided treatment decisions. Figure 2 illustrates the echocardiographic classification and its clinical implications.

Relevant findings and management decisions by patient

Patient 1 had extensive thrombi involving both the right and left heart chambers. Imaging confirmed Type B thrombi. Surgical embolectomy was deemed necessary due to the thrombus' obstructive potential. Despite the procedure, this patient succumbed to postoperative complications, underscoring the complexity of managing dual-chamber involvement.

Patient 2, diagnosed with APS, presented with dyspnea and bilateral pulmonary artery involvement. A mobile thrombus in the right atrium (RA) was identified as Type A. Systemic thrombolysis with alteplase resolved the thrombus, reflecting the efficacy of thrombolysis for highly mobile thrombi.

Patient 3 reported hemoptysis and was found to have a Type A thrombus in the right ventricle (RV). Management with direct oral anticoagulants (DOACs) stabilized the condition, highlighting the role of anticoagulation in intermediate-risk patients (Table 4).

**Table 4:** Absolute and Relative Contraindications to Systemic Thrombolysis. This table outlines the contraindications to the use of systemic thrombolysis in patients with CIT and PE, providing critical information for assessing the suitability of thrombolytic therapy. CIT, clot-in-transit; PE, pulmonary embolism

Absolute contraindications	Relative contraindications
Active bleeding	Age > 65 years, especially >75 years
Ischemic stroke in three months	Systolic blood pressure > 180 mmHg
Structural intracranial abnormalities such as a neoplasm, vascular malformation, or aneurysm	Diastolic blood pressure > 110 mmHg
Recent head trauma with fracture or brain damage	Ischemic stroke > three months
Current brain or spinal surgery	Recent major surgery, invasive procedures, and trauma
History of intracranial bleeding	Current pregnancy or childbirth (one week)

Patient 4 experienced respiratory distress requiring mechanical ventilation. Imaging revealed a Type A thrombus in the RA. Systemic thrombolysis led to clinical improvement, demonstrating the urgency of reperfusion strategies in high-risk CIT cases.

Patient 5, the youngest in the cohort, also had APS and bilateral pulmonary involvement. Anticoagulation successfully managed the condition, suggesting that well-selected cases of Type B thrombi may not require aggressive interventions.

Management strategies

The analysis of this case series highlights the variability in treatment approaches for CIT. Among the five cases, two patients underwent systemic thrombolysis, two received direct oral anticoagulants (DOACs), and one required surgical embolectomy. The selection of therapy was largely dictated by thrombus mobility, thrombus burden, and underlying risk factors such as APS or malignancy. Notably, while systemic thrombolysis effectively resolved mobile thrombi in high-risk patients, surgical embolectomy was required in one case with extensive thrombi involving multiple cardiac chambers. These findings underscore the importance of individualized risk stratification in CIT management.

CIT management lacks standardized guidelines due to its rarity. The literature suggests anticoagulation as the cornerstone therapy, particularly for intermediate-risk patients [12]. However, systemic thrombolysis remains the preferred option for high-risk cases, despite bleeding risks [13,14]. Recent studies highlight the complementary benefits of these interventions, particularly for mobile thrombi at high risk of embolization [2,15]. Surgical embolectomy, while invasive, provides a definitive solution for large or obstructive thrombi. In this cohort, the selection of treatment modalities, from anticoagulation to surgical interventions, was dictated by thrombus mobility, patient risk stratification, and comorbidities.

Emerging catheter-directed therapies, including mechanical thrombectomy and catheter-directed thrombolysis, offer minimally invasive alternatives, reducing clot burden with fewer bleeding complications (Table 4) [16]. A notable example includes the use of the Inari FlowTriever catheter under echocardiographic guidance, which has demonstrated promising results in recent case series [6].

Multidisciplinary collaboration and future directions

The integration of pulmonary embolism response teams (PERT) exemplifies the importance of multidisciplinary collaboration in CIT management. These teams enable rapid decision-making, particularly in dynamic clinical scenarios [17]. Future research should focus on developing standardized diagnostic criteria and treatment algorithms for CIT. Large-scale registries and randomized trials are essential to validate emerging therapies and optimize patient outcomes.

CIT in the context of PE demands tailored, multidisciplinary approaches that consider the advantages and disadvantages of CIT management. This case series, combined with a concise literature review, highlights the evolving landscape of CIT diagnosis and management. Continued research and innovation are imperative to improve the prognosis for this critical condition.

This case series reinforces that CIT management requires a tailored approach, balancing thrombus characteristics, patient comorbidities, and bleeding risk. While anticoagulation remains the mainstay for intermediate-risk patients, high-risk individuals may benefit from more aggressive strategies such as systemic thrombolysis or surgical embolectomy. Future studies are needed to establish standardized treatment guidelines for CIT, particularly regarding the role of emerging catheter-directed therapies.

## Conclusions

CIT is a rare but life-threatening manifestation of PE that requires prompt recognition and intervention. This case series highlights the clinical variability and management challenges of CIT, emphasizing the critical role of imaging in diagnosis and the need for individualized treatment strategies based on thrombus characteristics and patient-specific risk factors. While anticoagulation and systemic thrombolysis remain primary treatment options, surgical embolectomy and catheter-directed therapies provide essential alternatives in complex cases.

The findings underscore the importance of multidisciplinary collaboration and personalized approaches to optimize patient outcomes. Further large-scale studies and registry data are necessary to refine diagnostic criteria and establish standardized treatment protocols for CIT. Enhancing our understanding and management strategies will ultimately improve survival rates and reduce morbidity in this high-risk population.
